# What happens to qualitative studies initially presented as conference abstracts: A survey among study authors

**DOI:** 10.1017/rsm.2025.10033

**Published:** 2025-09-05

**Authors:** Marwin Weber, Simon Lewin, Joerg J. Meerpohl, Heather Menzies Munthe-Kaas, Rigmor Berg, Andrew Booth, Claire Glenton, Jane Noyes, Ingrid Toews

**Affiliations:** 1 Institute for Evidence in Medicine, Medical Center, University of Freiburg, Freiburg im Breisgau, Germany; 2 Medical Faculty, University of Freiburg, Freiburg im Breisgau, Germany; 3 Centre for Epidemic Interventions Research (CEIR), Norwegian Institute of Public Health, Oslo, Norway; 4 Department of Health Sciences in Ålesund, Norwegian University of Science and Technology (NTNU), Ålesund, Norway; 5 Health Systems Research Unit, South African Medical Research Council, Cape Town, South Africa; 6 Cochrane Germany, Cochrane Germany Foundation, Freiburg Germany; 7 Division of Health Services, Norwegian Institute of Public Health, Oslo, Norway; 8 UiT The Arctic University of Norway, Tromsø, Norway; 9 Sheffield Centre for Health and Related Research (SCHARR), Faculty of Health, University of Sheffield, Sheffield, UK; 10 Western Norway University of Applied Sciences, Bergen, Norway; 11 School of Health Science, Bangor University, Bangor, UK

**Keywords:** dissemination bias, GRADE, GRADE-CERQual, publication bias, qualitative research, survey

## Abstract

Qualitative research addresses important healthcare questions, including patients’ experiences with interventions. Qualitative evidence syntheses combine findings from individual studies and are increasingly used to inform health guidelines. However, dissemination bias—selective non-dissemination of studies or findings—may distort the body of evidence. This study examined reasons for the non-dissemination of qualitative studies. We identified conference abstracts reporting qualitative, health-related studies. We invited authors to answer a survey containing quantitative and qualitative questions. We performed descriptive analyses on the quantitative data and inductive thematic analysis on the qualitative data. Most of the 142 respondents were female, established researchers. About a third reported that their study had not been published in full after their conference presentation. The main reasons were time constraints, career changes, and a lack of interest. Few indicated non-publication due to the nature of the study findings. Decisions not to publish were largely made by author teams. Half of the 72% who published their study reported that all findings were included in the publication. This study highlights researchers’ reasons for non-dissemination of qualitative research. One-third of studies presented as conference abstracts remained unpublished, but non-dissemination was rarely linked to the study findings. Further research is needed to understand the systematic non-dissemination of qualitative studies.

## Highlights

### What is already known?

Dissemination bias, also known as publication bias, is a well-researched phenomenon in quantitative research that has led to poor and harmful decisions in healthcare. Dissemination bias might also impact qualitative research, but there is little evidence about it.

### What is new?

Our survey revealed that a considerable proportion of health-related qualitative studies remain unpublished 6–8 years after they were presented at a conference. The reasons for non-dissemination mainly relate to limited time and resources for publication. The study’s main message and findings were rarely a reason for non-dissemination.

### Potential impact for RSM readers

Researchers who conduct evidence syntheses and decision-makers may benefit from our study’s findings, as they inform about the mechanisms behind non-dissemination in qualitative research.

## 1 Background

There are many essential research questions in healthcare that cannot be tackled with quantitative research methods. These include patients’ experiences with and views of a certain health intervention. For such questions, qualitative methods are a suitable methodological approach. Findings from qualitative research on these questions can inform decisions in evidence-based healthcare and contribute to policy decisions. A key benefit of the use of qualitative evidence is representing the views of a wide range of stakeholders in decision-making, including vulnerable and marginalized groups.^1^

Qualitative evidence syntheses (QESs) are a way of synthesizing evidence from individual qualitative studies.^2^ The resultant composite findings are used increasingly in health guidelines.^3^ ^–^ ^5^ The Grading of Recommendations Assessment, Development and Evaluation-Confidence in the Evidence from Reviews of Qualitative research (GRADE-CERQual) approach^6^ ^–^ ^11^ is widely used to assess how much confidence to place in findings from QES.^12^ Dissemination bias was originally proposed as a key component of the GRADE-CERQual approach based on its importance in the GRADE approach for quantitative research.^13^ However, this component is currently not included in GRADE-CERQual.

Dissemination bias (often referred to as publication bias) describes a systematic error occurring from the systematic non-dissemination of studies and individual findings. In the context of qualitative research, the GRADE-CERQual working group defines it as “a systematic distortion of the phenomenon of interest due to selective dissemination of qualitative studies or the findings of qualitative studies”.^13^ Around 50% of quantitative clinical effectiveness research remains unpublished once completed.^14^ The non-dissemination is often associated with unfavorable results.^15^ As a consequence, the resultant dissemination bias leads to an overestimation of the reported effects of health interventions in evidence synthesis.^16^ In the past, this has led to the implementation of ineffective and harmful health interventions, as well as research waste.^17^ ^,^ ^18^ While the impacts of dissemination bias are well-researched in quantitative research,^19^ ^–^ ^21^ very little is known about this phenomenon in qualitative research.

Previous research found that around one-third of qualitative studies that were presented as conference abstracts were not subsequently disseminated in full.^22^ ^,^ ^23^ Underlying reasons for the non-dissemination mainly revolve around resources and quality^24^ and seem to be similar in both qualitative and quantitative research. There is a research gap with regard to understanding the possibility of dissemination bias in qualitative research that needs to be addressed. This survey aimed to examine reasons for the non-dissemination of qualitative studies. Specifically, we wanted to find out whether non-dissemination might be connected to the findings of a study.

## 2 Methods

This study is part of a larger research project,^25^ aimed at quantifying the proportions of and exploring the reasons for non-dissemination in qualitative research by tracking a cohort of conference abstracts to their full publication. In this sub-study, we used a cross-sectional survey design. We report the study in accordance with the Consensus-Based Checklist for Reporting of Survey Studies ^26^ guideline. We followed an internal study protocol that can be requested from the corresponding author.

### 2.1 Participant selection

Study participants were authors of conference abstracts. To recruit participants, we first identified conference abstracts reporting qualitative, health-related research studies through a search in the Web of Science Conference Proceedings Citation Index—Science (CPCI-S) and the Conference Proceedings Citation Index—Social Science & Humanities (CPCI-SSH) via the Web of Science Platform (Clarivate) on August 10, 2023. In order to determine the studies’ publication status, we contacted the study authors of these conference abstracts, thereby drawing a single stage convenience sample. The authors’ contact details were partially included in the conference abstract record in the database or were obtained via online searches and were subsequently used to send study invitations. In case the contacted authors were unavailable or not interested in taking the survey, we asked them to forward the survey to other relevant researchers.

### 2.2 Data collection tool

The English-language survey consisted of a total of 19 questions subdivided into four sections:

1: Details about the participants’ conference abstract and availability of subsequent publications.

2: Reasons for non-dissemination of the participants’ conference abstract.

3: Details about non-dissemination and dissemination bias in the participants’ research overall.

4: Demographic information.

Fourteen of the questions were single or multiple choice; seven of these asked for optional free-text elaborations and comments. There were two multiple-choice grids with 5-point Likert scales, also including a free-text option to elaborate and comment. Three questions asked for free-text responses. Multiple responses to multiple-choice questions were counted equally. The survey was customized for each respondent using intra-survey filters that adjusted based on their answers to previous questions. This approach minimized respondent burden and enhanced the relevance of their responses. The survey, including invitation and participant information, is available in Supplementary Material 1.

We shared the survey for review and feedback among all co-authors. After revisions, we shared it for pretesting among 11 researchers from the authors’ professional networks, all of whom had experience in conducting and reporting health-related qualitative research and were familiar with the concept of systematic non-dissemination. This might partially distinguish them from the target population of the survey. Five researchers provided comments through the software’s piloting function, and we revised the survey according to their feedback to achieve a maximum validity and reliability in each question. We created the survey in the SoSci Survey software.^27^ We maintained gender neutral language in the survey.

### 2.3 Survey administration

Participants were invited to complete the survey online from February 6, 2024 to April 3, 2024. We sent the first survey invitation and four reminders at 2-week intervals via personalized serial emails through the SoSci Survey platform. Non-functional email addresses of authors were initially followed up through Google searches, and the invitation was forwarded to the authors’ new contact details. However, because the majority of the newly retrieved email addresses also proved to be non-functional, we did not pursue a complete follow-up of all non-functional addresses. The contacted authors could access the survey through a personalized link provided in the email to prevent multiple participation. This link allowed for single participation per abstract.

### 2.4 Data protection and ethics

We discussed data protection concerns in the context of the survey with the data protection entity of the University Medical Centre Freiburg, and no concerns were raised in connection with the survey. The ethics review board of the University of Freiburg approved the study (22-1528). Participation in the survey was voluntary and could be discontinued at any time. No personal data were requested in any of the questions. All data and answers were stored in a password-protected, institutional server environment. We collected, processed, and analyzed all data in an aggregated form. We informed the participants of the purpose of our survey and explained our data protection concept.

### 2.5 Data extraction and analyses

The survey platform allowed all data to be downloaded directly into CSV and MS Excel files. We used this function for data extraction and preparation of the data for analysis. We analyzed quantitative data with a descriptive statistical analysis in MS Excel. The responses in text format were processed and coded in an inductive thematic analysis in six steps^28^ ^–^ ^30^ by one researcher (IT) and checked by a co-author (MW).

We included all available responses from participants who completed one or more questions in the analysis. Results are reported separately for each question, based on the number of responses, and proportions were calculated accordingly.

## 3 Results

### 3.1 Participant characteristics

We sent 1097 survey invitations. About 250 non-functional emails bounced. Exact figures on non-responses caused by non-functional email addresses and spam filters could not be determined. Overall, 142 people initiated the survey, and 130 people (91.5%) completed the survey. The resulting response rate ranges between 12.9% and 18.9%. In brief, the respondents—authors of conference abstracts of qualitative research—as shown in Table 1, were largely female (77.2%), established or leading researchers (69.2%), and over 40 years of age (74.4%). Their main region of work was primarily Europe or North America (82.4%), and their main type of affiliation was a higher education institution or public research institute (88.4%).

**Table 1:** Description of the study participant characteristics

Characteristic	*N*	%
Career level (*n* = 130)		
First stage researcher	12	9.23
Recognized researcher	23	17.69
Established researcher	67	51.54
Leading researcher	23	17.69
Prefer not to say	5	3.85
Gender (*n* = 123)		
Male	95	77.24
Female	27	21.95
Prefer not to say	1	0.81
Age (*n* = 129)		
16–20	1	0.78
21–25	0	0
26–30	1	0.78
31–35	12	9.30
36–40	19	14.73
41–45	32	24.81
46–50	26	20.16
51–65	31	24.03
65+	7	5.43
Main region of work (*n* = 125)		
Europe	59	47.2
North America	44	35.2
South America	1	0.80
Africa	1	0.80
Asia	16	12.8
Australia and Oceania	4	3.20
Type of main affiliation (*n* = 130)		
Higher education institution	100	76.92
Public research institute	15	11.54
Government or ministry institution	4	3.08
I do mainly freelance work	3	2.31
Private research institute/foundation	3	2.31
Research department of a private company	1	0.77
Prefer not to say	3	2.31

### 3.2 Extent of non-dissemination of conference abstracts

Of the 139 participants who responded to this question, the majority, 100 (71.9%) indicated that their study had been published in full after presentation at the conference, while 39 (28.1%) reported that their study had not (one respondent explained that they were still preparing the full publication). Two participants did not respond to the respective survey question but provided a link to the full publication of their work via email.

#### 3.2.1 Reasons for non-publication of studies

**Figure 1:**
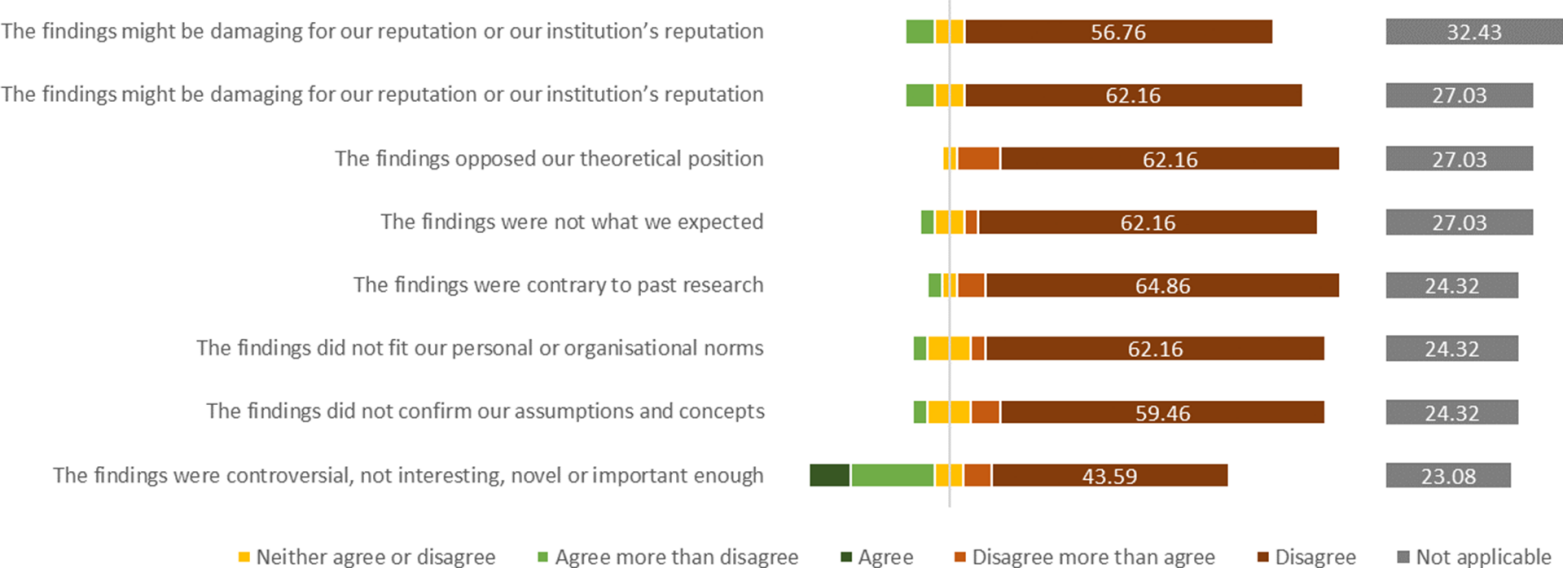
Participants’ level of agreement with reasons for the non-dissemination of their study presented at a conference.

Most participants who reported that the study presented as a conference abstract was not published disagreed that the non-publication was connected to the study’s findings (Figure 1).

In participants’ free-text responses, a common theme was insufficient time to pursue the publication of the study. In two instances, the main investigator had conducted the study as part of their Master’s degree project, had moved on in their career, and did not publish the study. Two other respondents said that the publication was still planned. Single participants reported that the sample size was low, that the study was never intended for publication, or that the study was not published because the publication was not a priority. One participant wrote “The findings were not so interesting, to be published in a full paper” (Case 184), and another one stated that the reviewers found the time gap between study conduct and submission too long for a publication (Case 340).

When asked about who made the decision about the study’s non-publication, the participants’ responses (*n* = 32) showed that different stakeholders were involved in the decision-making. Only nine participants indicated that multiple stakeholders (two or more) were involved in the decision (Table 2).

**Table 2:** Stakeholders involved in the publication decision (multiple responses possible)

Who mainly decided that…	…the *study* should not be published (multiple answers possible) (*N* = 32)	…the *finding(s)* should not be published (*N* = 12)
Survey participant/researcher	15	6
Co-authors	12	5
Peer-reviewers	2	2
Editors	3	2
Sponsors	0	0
Funders	1	0
Others	12	2

In free-text responses, one participant reported that the principal investigator made the decision, another one highlighted the supervisors’ role by reporting: “The supervisor did not show any interest in getting it either published or extend the findings” (Case 305), while a third stated that the “location of research” was critical for the publication decision (Case 171). One participant stated that the decision was made by the authors themselves. No further details were reported to explain the latter.

#### 3.2.2 Reasons for non-publication of individual findings

Of those who reported that the study that they had presented as a conference abstract *was* published (*n* = 100/71.9%), 12 participants indicated that some important findings were not included, while 84 participants said that all important findings were included. The remaining participants ended their participation before answering this question.

**Figure 2:**
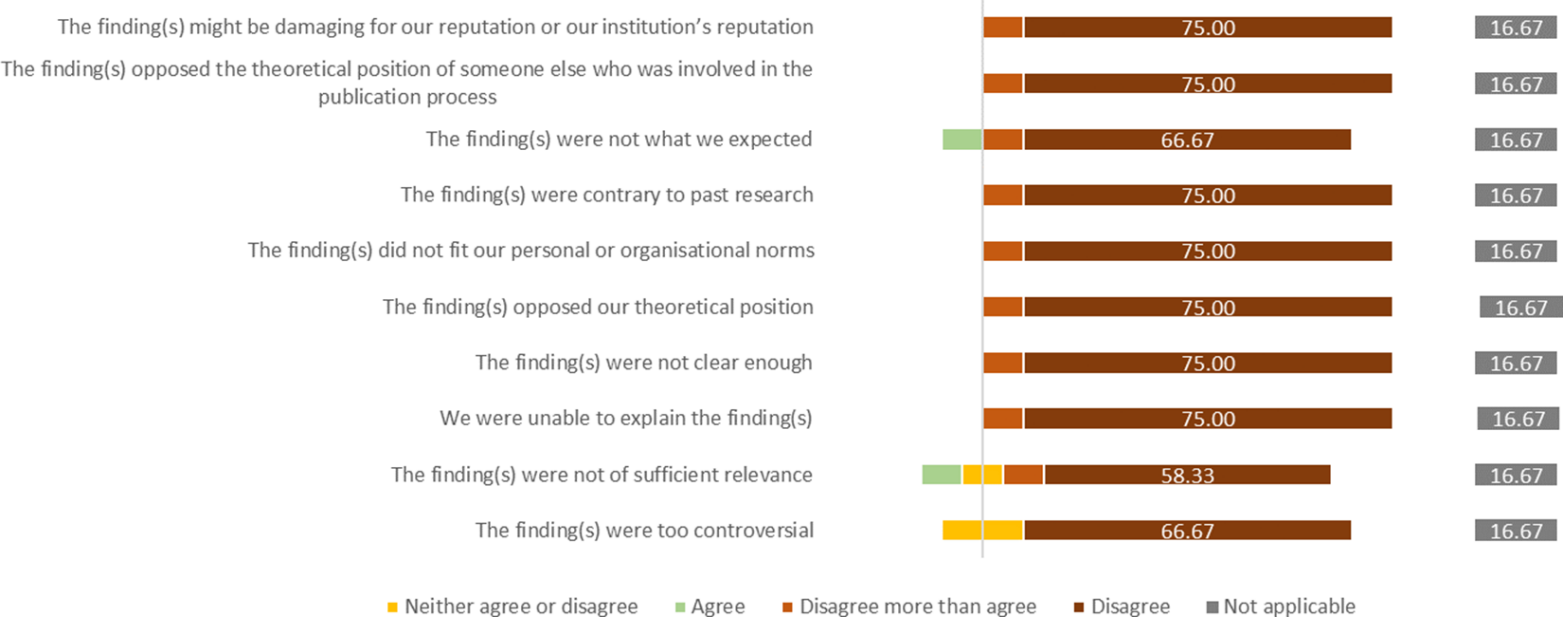
Participants’ level of agreement with reasons for the non-dissemination of any of their qualitative studies.

Among those 12 respondents who answered that important findings were missing from the full text publication, most responded that they disagreed that the non-publication was related to the nature and content of their findings (see Figure 2).

All these 12 participants gave more details in free-text responses. A frequently occurring theme was the space limitation set by journals and time constraints. Others said that the publication of important findings was still planned or not intended in the first place. A few respondents reported that the findings were split in multiple papers because the results “became way to complex” (Case 148) and that “Another publication was written (in English) including all the results […], however the pandemic hit […] and the publication was deemed not enough relevant to the pandemic context” (Case 98).

### 3.3 Non-dissemination of qualitative studies and individual findings in general

When asked about all of their qualitative work, 24 (18.5%) answered that “some studies remained unpublished.” This was mainly decided by the survey respondent themselves (*n* = 10) and/or co-authors (*n* = 9), peer-reviewers (*n* = 4), editors (*n* = 4), sponsors, (*n* = 1), funders (*n* = 1), or others (*n* = 7) (multiple responses possible).

Eight participants said that they had removed individual findings from a report due to their nature and content. They elaborated that the findings were not relevant enough or revealed no new knowledge. In another case, the whole paper was too extensive, so it needed to be shortened, and certain findings were dropped or moved to a supplement. The survey participants did not report other details about the nature and content of findings that were omitted. In two instances, the respondent was involved in the decision about which findings to publish. Co-authors (*n* = 5), peer-reviewers (*n* = 3), and editors (*n* = 1) were also mentioned as being involved in the publication decision.

## 4 Discussion

### 4.1 Summary

This study provides a comprehensive and pioneering overview of researchers’ reasons for non-dissemination in qualitative research. Through a survey conducted among researchers in health and healthcare, we found that one-third had not published a qualitative study they had originally presented as a conference abstract. Participants reported that the decision to publish was generally not influenced by the study’s message or findings. In most cases, the decision to publish was made by the main authors themselves. Among those who did publish their qualitative study, only half reported that all results were included in the final publication.

In contrast to quantitative research, where non-dissemination due to the direction of the results is a documented problem,^15^ we found in this author survey that the non-dissemination of qualitative research was rarely influenced by the findings or message of the studies. This indicates that other factors common to both qualitative and quantitative research, such as lack of time and resources, are more likely to contribute to the non-dissemination of qualitative research.^31^ The survey also revealed that around a third of the qualitative studies examined remained unpublished. A similar proportion of unpublished studies was identified in the larger research project to which this survey belongs. Moreover, for one-third of the studies that were followed-up in another study, no full publication could be located through searches in electronic databases or grey literature.^23^ Generally, the rate of non-dissemination in qualitative research was found to range from 68.1%^24^ to 55.8%^22^ to 33.7%.^23^

### 4.2 Strength and limitations

Self-reported data seem to be the only feasible way to gather data on authors’ reasons for non-dissemination, especially in qualitative research. This survey provided an efficient method for collecting international data on a widely relevant topic. However, its design bears some limitations that could impact the validity and interpretability of the findings, including hesitancy to participate, selection bias, recall bias, and social desirability bias. To address these challenges, the study incorporated several features to enhance both participation and the truthfulness of the responses: (1) Testing and optimizing the survey design. The survey was made as comprehensive and simultaneously as user-friendly as possible, by conducting pretests with cooperation partners and qualitative researchers. Feedback was carefully reviewed, and the survey, along with the participant materials, was revised to ensure clarity, brevity, and inclusion of well-designed questions. As a result, 91.5% of those who took the survey completed it. Furthermore, to minimize respondent burden, the survey employed intra-survey filters that adjusted based on their answers to previous questions, thus tailoring the survey questions to each participant. (2) Increasing the survey accessibility. The survey was hosted on a platform optimized for various devices and operating systems, ensuring compatibility with PCs, tablets, and smartphones. (3) Assuring anonymity of the participants. Anonymity was guaranteed during data processing, analysis, and reporting. Data handling procedures were discussed with the institutional data protection officer, and this assurance was communicated to the participants in the invitation and information materials. (4) Increasing motivation to participate. The study’s aim was clearly explained in the invitation message and the participant information. A modest incentive, such as updates on the research process and outcomes, was offered to encourage participation. (5) Balanced and careful interpretation of findings. We reported results and interpreted them with balance and caution, taking into account the limitations of the study design.^26^ ^,^ ^32^

Still, the response rate was relatively low at an estimated maximum of 18.9%. One potential reason for this is the extended follow-up period, as we sampled studies from conferences held between 2016 and 2018. Inevitably, researchers were unreachable due to changed or outdated contact details. This follow-up duration was necessary to allow sufficient time for the full publication of the studies. In addition, the response rate was likely affected by the substantial number of non-functional or bounced email addresses. The challenge was likely particularly relevant for projects led by primary authors in their qualification phase (student projects). While the proportion of student projects could not be quantified, and most survey participants reported that they were established researchers, we anticipate that a sizeable proportion of studies presented at conferences were led by individuals in the early stages of their academic careers.

A comparison of the survey participants’ characteristics with those of the authors of the larger study revealed similarities with regard to gender and geographic region of their affiliation. However, there was insufficient data available to compare other characteristics.

### 4.3 Future research

To expand our understanding of the systematic non-dissemination of qualitative studies based on their finding or messages, further research is needed. Case studies highlighting instances of non-dissemination, whether of entire studies or individual findings, would provide valuable insights into the pathways leading to systematic non-dissemination or dissemination bias. More qualitative research, such as interviews or focus group discussions, could offer in-depth accounts of researchers’ experiences with dissemination bias. Furthermore, it may be valuable to consider whether and how funding sources and potential conflicts of interest could influence the full dissemination of qualitative research findings. Although funding bias has primarily been investigated and discussed in the context of quantitative research,^33^ it may also be relevant in qualitative research^34^ and thus warrants consideration in future research on dissemination bias. Such insights would support the development of a framework to better understanding dissemination bias in qualitative research, its underlying causes, and its potential impact on healthcare and decision-making. Ultimately, this would pave the way for implementing measures to reduce and prevent dissemination bias.

## 5 Conclusions

This study offers an overview of researchers’ self-reported experiences with the non-dissemination of qualitative studies and individual findings due to the nature and content of their results. Our findings are novel in their relevance for future in-depth investigations, including case studies on actual dissemination bias in qualitative research, and for contributing to a broader understanding of the potential for dissemination bias in qualitative research. The plausibility of this bias should be considered when assessing the confidence we place in findings from QESs used for decision-making.^13^

## Supporting information

Weber et al. supplementary material 1 Weber et al. supplementary material

Weber et al. supplementary material 2 Weber et al. supplementary material

## Data Availability

Data are available as a supplement to the manuscript publication.

## Supplementary material

To view supplementary material for this article, please visit http://doi.org/10.1017/rsm.2025.10033.
