# Supplementary material for: What happens to qualitative studies initially presented as conference abstracts: A survey among study authors
Source: Res Synth Methods. 2025 Sep 5;16(6):1025–34. doi: 10.1017/rsm.2025.10033 (PMC12657646; doi:10.1017/rsm.2025.10033)
Supplement: Weber et al. supplementary material 1 — Weber et al. supplementary material [file S1759287925100331sup001.pdf]

soSci
oFb - der onlineFragebogen

GRADE-CERQual → base 17.07.2024, 11:01

Participant information

Welcome and thank you very much for taking the time to participate. This survey will take approximately **5 to 10 minutes** of your time.

Why you?

We identified you as an author of a conference abstract that describes a qualitative study. We would like to have more information about this particular conference abstract and your experience in publishing qualitative studies. This is because we want to understand possible 'dissemination bias' in qualitative health research.

What is our aim?

Dissemination bias (also referred to as 'publication bias') happens when certain types of research findings are more likely to be published and disseminated than others. We know a lot about dissemination bias in quantitative research. But we don't know much about this phenomenon in qualitative research. We are therefore following up a set of conference abstracts focused on qualitative research to see what happened to these studies, and to try to understand why some studies are published as full papers and others are not.

What you will have to do

The following questionnaire contains a series of questions about your qualitative research study presented as conference abstract. You are asked to answer them by selecting answer option or adding free text responses according to your personal opinion and experience. The survey is conducted exclusively online.

What are the benefits of participation?

With your participation, you are making a valuable contribution to our research. Upon your request, we will be happy to send you the results after the completion of our study.

What are the risks?

We don't expect there to be any risks linked to your participation. There are no costs for you as a study participant.

Privacy and confidentiality

We will take all necessary measures that the data collected and further processed for scientific purposes cannot and will not be assigned to you as a person. All data will be coded and analysed in aggregate form and it will not be possible to trace it back to individual participants. The data will be stored for 5 years on password-protected servers at the University of Freiburg. The survey will be conducted using the SoSci Survey portal (https://www.soscisurvey.de). The portal takes the following data protection aspects into account: SSL encryption, no storage of IP addresses in log files, no cookies, server location in Munich. More detailed information can be found at https://soscisurvey.de/de/privacy.

Participation in the survey is voluntary. You can terminate your participation in this survey at any time and without giving reasons, without incurring any disadvantages. To do so, simply close the browser window of the

survey.

The study is in line with the Declaration of Helsinki. In addition, all data protection requirements of the EU General Data Protection Regulation (DSGVO) are complied with. Deletion of the data is possible upon request until the data has been included in the evaluation. Afterwards, the data is left anonymous in the evaluation.

The study was reviewed by the Research Ethics Committee of the University of Freiburg (22-1528).

Questions and remarks are very welcome at: Ingrid Toews (study coordinator) ingrid.toews@uniklinik-freiburg.de | +49 761 – 270 85332 | Institute for Evidence in Medicine | Breisacher Straße 86 | DE-79110 Freiburg

Definitions

Qualitative research - aims to explore how people perceive and experience the world around them. Qualitative researchers typically rely on interviews, documents or observation to explore people's perceptions and experiences in connection with their health and with the use of health-care services. They then explore the data by means of qualitative analytical methods and present their findings narratively rather than through numbers. (Glenton and Lewin 2014)

Dissemination bias in qualitative research - systematic distortion of the phenomenon of interest due to selective dissemination of studies or individual findings of studies.

Page 02

A101

As mentioned in our email, we have identified your conference abstract and would like to find out more about the publication or dissemination of your research.

A102

1. In what format did you present your study at the conference?

- ☐ Traditional poster (printed on paper)
- ☐ Video poster
- ☐ Live oral presentation
- ☐ Recorded oral presentation
- ☐ Other

A103

2. Were any of the study findings reported in the conference abstract made available as a scientific publication or a report in the grey literature* at any time following the conference?

*Grey literature refers to research produced outside of the traditional commercial or academic publishing and distribution channels.

- ○ Yes
- ○ No

11 Active Filter(s)

Filter A103/F1
If any of the following options is selected: **2**
Then hide question/text **A202** placed later in the questionnaire

Filter A103/F2
If any of the following options is selected: **2**
Then hide question/text **A203** placed later in the questionnaire

Filter A103/F3
If any of the following options is selected: **1**
Then hide question/text **A301** placed later in the questionnaire

Filter A103/F4
If any of the following options is selected: **1**
Then hide question/text **A302** placed later in the questionnaire

Filter A103/F5
If any of the following options is selected: **1**
Then hide question/text **A303** placed later in the questionnaire

Filter A103/F6
If any of the following options is selected: **2**
Then hide question/text **A401** placed later in the questionnaire

Filter A103/F7
If any of the following options is selected: **2**
Then hide question/text **A501** placed later in the questionnaire

Filter A103/F8
If any of the following options is selected: **2**
Then hide question/text **A502** placed later in the questionnaire

Filter A103/F9
If any of the following options is selected: **2**
Then display question/text **A903** placed later in the questionnaire (otherwise hide)

Filter A103/F10
If any of the following options is selected: **1**
Then hide question/text **A304** placed later in the questionnaire

Filter A103/F11
If any of the following options is selected: **2**
Then hide question/text **A503** placed later in the questionnaire

---

Page 03

A203

3. Can you please share the reference(s) (or URL or doi or file) for the scientific publication or full report of your study?

To identify the doi number for a published work you can type the title into the “search Metadata” tab of the www.crossref.org/ site

A202

Durchsuchen... Keine Datei ausgewählt.

A301

Dissemination practice in qualitative research

The next few questions ask about your practical experiences in disseminating the findings of the qualitative research study mentioned in our email.

A302

4. There are many reasons why research is not published. Do any of the following reasons apply to your study?

	Agree	Agree more than disagree	Neither agree or disagree	Disagree more than agree	Disagree	Not applicable
The findings were controversial, not interesting, novel or important enough	○	○	○	○	○	○
The findings did not confirm our assumptions and concepts	○	○	○	○	○	○
The findings did not fit our personal or organisational norms	○	○	○	○	○	○
The findings were contrary to past research	○	○	○	○	○	○
The findings were not what we expected	○	○	○	○	○	○
The findings opposed our theoretical position	○	○	○	○	○	○
The findings opposed the theoretical position of someone else who was involved in the publication process	○	○	○	○	○	○
The findings might be damaging for our reputation or our institution's reputation	○	○	○	○	○	○

A303

○ Other reasons (please elaborate)

A304

4.1 Who mainly decided that your study should not be published?

Select all that apply

- [ ] I decided this
- [ ] Co-authors
- [ ] Peer-reviewers
- [ ] Editors
- [ ] Sponsors, carrying the main responsibility for the study by planning and conducting the study and acquiring funding for the study
- [ ] Funders, providing funding for the study
- [ ] Others (please elaborate)

Page 05

A401

5. Did the scientific publication(s) or full report(s) of your study include all individual findings that you (and your co-authors) considered important?

- ( ) Yes, all important findings were included
- ( ) No, some important findings were not included

4 Active Filter(s)

Filter A401/F1
If any of the following options is selected: **1**
Then hide question/text **A501** placed later in the questionnaire

Filter A401/F2
If any of the following options is selected: **1**
Then hide question/text **A502** placed later in the questionnaire

Filter A401/F3
If any of the following options is selected: **2**
Then display question/text **A903** placed later in the questionnaire (otherwise hide)

Filter A401/F4
If any of the following options is selected: **1**
Then hide question/text **A503** placed later in the questionnaire

Page 06

A501

6. If you did disseminate your study as a scientific publication or full report, but did not include all important findings, do you agree with the following reasons why specific important study findings were not included in your reports or papers?

	Agree	Agree more than disagree	Neither agree nor disagree	Disagree more than agree	Disagree	Not applicable
The finding(s) were too controversial	Agree	Agree more than disagree	Neither agree nor disagree	Disagree more than agree	Disagree	Not applicable
The finding(s) were not of sufficient relevance	Agree	Agree more than disagree	Neither agree nor disagree	Disagree more than agree	Disagree	Not applicable
We were unable to explain the finding(s)	Agree	Agree more than disagree	Neither agree nor disagree	Disagree more than agree	Disagree	Not applicable
The finding(s) were not clear enough	Agree	Agree more than disagree	Neither agree nor disagree	Disagree more than agree	Disagree	Not applicable
The finding(s) opposed our theoretical position	Agree	Agree more than disagree	Neither agree nor disagree	Disagree more than agree	Disagree	Not applicable
The finding(s) did not fit our personal or organisational norms	Agree	Agree more than disagree	Neither agree nor disagree	Disagree more than agree	Disagree	Not applicable
The finding(s) were contrary to past research	Agree	Agree more than disagree	Neither agree nor disagree	Disagree more than agree	Disagree	Not applicable
The finding(s) were not what we expected	Agree	Agree more than disagree	Neither agree nor disagree	Disagree more than agree	Disagree	Not applicable
The finding(s) opposed the theoretical position of someone else who was involved in the publication process	Agree	Agree more than disagree	Neither agree nor disagree	Disagree more than agree	Disagree	Not applicable
The finding(s) might be damaging for our reputation or our institution's reputation	Agree	Agree more than disagree	Neither agree nor disagree	Disagree more than agree	Disagree	Not applicable

A502

○ Other (please specify) ____________

A503

6.1 Who decided that the finding(s) should not be published?

Select all that apply

- ☐ I decided this
- ☐ Co-authors
- ☐ Peer-reviewers
- ☐ Editors
- ☐ Sponsors, carrying the main responsibility for the study by planning and conducting the study and acquiring funding for the study
- ☐ Funders, providing funding for the study
- ☐ Others (please elaborate) ____________

Page 07

A601

Dissemination bias in qualitative research

The following questions apply to ALL of your qualitative work and not just the work presented in the conference abstract

A602

7. Have any of your qualitative studies not been published because of their findings? If yes, please copy and paste your study aim or research questions here.

If there is more than one study involved here, please indicate different studies by numbers. For example, "Study 1: ...."

- ○ No, all studies are published
- ○ Yes, some studies remained unpublished (please specify) ____________

4 Active Filter(s)

Filter A602/F1
If any of the following options is selected: **1**
Then hide question/text **A701** placed later in the questionnaire

Filter A602/F2
If any of the following options is selected: **1**
Then hide question/text **A702** placed later in the questionnaire

Filter A602/F3
If any of the following options is selected: **1**
Then hide question/text placed later in the questionnaire

Filter A602/F4
If any of the following options is selected: **2**
Then display question/text **A903** placed later in the questionnaire (otherwise hide)

Page 08

A701

8. Who mainly decided that the study/studies addressed in the previous question should not be published?

Select all that apply

- [ ] I decided this
- [ ] Co-authors
- [ ] Peer-reviewers
- [ ] Editors
- [ ] Sponsors, carrying the main responsibility for the study by planning and conducting the study and acquiring funding for the study
- [ ] Funders, providing funding for the study
- [ ] Others (please elaborate) ________

Page 09

A801

9. Have individual findings ever been removed from any of your qualitative publications during the journal or report editorial process because of the nature and/or content of these findings? If yes, please tell us more about the findings that were omitted and if possible provide details about the study.

- ○ No
- ○ Yes (please elaborate) ________

3 Active Filter(s)

Filter A801/F1
If any of the following options is selected: **1**
Then hide question/text **A901** placed later in the questionnaire

Filter A801/F2
If any of the following options is selected: **1**
Then hide question/text **A902** placed later in the questionnaire

Filter A801/F3
If any of the following options is selected: **1**
Then hide question/text **A903** placed later in the questionnaire

Page 10

A901

10. Who decided that the finding(s) should not be published?

Select all that apply to the studies you referred to in the previous question.

- [ ] I decided this
- [ ] Co-authors
- [ ] Peer-reviewers
- [ ] Editors
- [ ] Sponsors, carrying the main responsibility for the study by planning and conducting the study and acquiring funding for the study
- [ ] Funders, providing funding for the study
- [ ] Others (please elaborate) __________

A903

We would like to follow up on your experiences with studies that remained unpublished or publications where important findings are missing. If you are willing to be contacted directly, please tick the box below.

- ○ Yes
- ○ No

B001

General Information

B002

11. What is your age?

[Please choose]

B003

12. What is your sex?

[Please choose]

B005

14. Please indicate which of the four career stages describes your current position best.

- First Stage Researcher (up to the point of PhD)
- Recognised Researcher (PhD holders or equivalent who are not yet fully independent)
- Established Researcher (researchers who have developed a level of independence)
- Leading Researcher (researchers leading their research area or field)
- Prefer not to say

B006

15. What best describes the institution to which you are mainly affiliated?

- Higher education institution
- Government or ministry institution
- Public research institute
- Private research institute / foundation
- Research department of a private company
- I do mainly freelance work
- Prefer not to say

B007

16. Where is the institution based that you are mainly affiliated with?

Please add at least one country.

Page 12

B101

18. Do you have any other comments about non-dissemination and dissemination bias in relation to your qualitative research?

B102

You have completed the survey! Thank you very much. Your participation will certainly help in answering our research aims.

If you are not willing to participate in an in-depth interview on this topic, please tick the box below.

B103

- [ ] I do not want to participate in any follow up research

Last Page

You have completed the survey!

Your answers were transmitted, you may close the browser window or tab now.

Ingrid Toews, Institute for Evidence in Medicine – 2022
